# Wnt signaling in synaptogenesis of Alzheimer's disease

**DOI:** 10.1002/ibra.12130

**Published:** 2023-09-06

**Authors:** Cheng‐Ting Zhang, Joy Wang, Wen‐Yuan Wang

**Affiliations:** ^1^ Living Systems Institute University of Exeter Exeter UK; ^2^ Winchester High School Winchester Massachusetts USA; ^3^ Interdisciplinary Research Center on Biology and Chemistry Shanghai Institute of Organic Chemistry, Chinese Academy of Science Shanghai China; ^4^ Huashan Hospital Fudan University Shanghai China

**Keywords:** AD, dendritic filopodia, synaptogenesis, Wnt

## Abstract

Alzheimer's disease (AD), recognized as the leading cause of dementia, occupies a prominent position on the list of significant neurodegenerative disorders, representing a significant global health concern with far‐reaching implications at both individual and societal levels. The primary symptom of Alzheimer's disease is a decrease in synaptic potency along with synaptic connection loss. Synapses, which act as important linkages between neuronal units within the cerebral region, are critical in signal transduction processes essential to orchestrating cognitive tasks. Synaptic connections act as critical interconnections between neuronal cells inside the cerebral environment, facilitating critical signal transduction processes required for cognitive functions. The confluence of axonal and dendritic filopodial extensions culminates in the creation of intercellular connections, coordinated by various signals and molecular mechanisms. The progression of synaptic maturation and plasticity is a critical determinant in maintaining mental well‐being, and abnormalities in these processes have been linked to the development of neurodegenerative diseases. Wnt signaling pathways are important to the orchestration of synapse development. This review examines the complicated interplay between Wnt signaling and dendritic filopodia, including an examination of the regulatory complexities and molecular machinations involved in synaptogenesis progression. Then, these findings are contextualized within the context of AD pathology, allowing for the consideration of prospective therapeutic approaches based on the findings and development of novel avenues for future scientific research.

## Graphical abstract

Alzheimer's disease (AD) is the leading etiological factor in the development of dementia. A prominent causative factor for its onset is the aggregation of amyloid β‐protein (Aβ). The presence of Aβ aggregates contributes to abnormal axonal and presynaptic terminal functioning, ultimately resulting in the formation of immature synapses connecting presynaptic terminals with a variety of dendritic spines. This process is potentially modulated by regulatory mechanisms orchestrated by the Wnt signaling pathway.

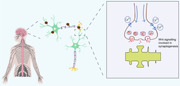

## 1 INTRODUCTION

Neurodegenerative diseases are uncommon hereditary and sporadic disorders of the central nervous system (CNS) that result in a slow and cumulative loss of function of a particular population of neurons.^1^ Degenerative nervous system diseases pose significant public and medical health burdens on populations worldwide.^2^ Unlike metabolic or toxic diseases, neurodegenerative diseases are distinguished by a progressive loss of selectively vulnerable neuronal populations.^3^ The main clinical features of a neurodegenerative disease, as well as its anatomical distribution and substantial molecular abnormalities, can all be used to classify it. Amyloid lesions, α‐synucleinopathies, tauopathies, and TAR DNA‐binding protein (TDP‐43) lesions are the most common neurodegenerative diseases.^3^

Alzheimer's disease (AD), the leading cause of dementia, is a serious global health issue with far‐reaching consequences for individuals and society. The initial phase of the disorder is characterized by deficits in the ability to encode and store new memories. Later stages are then accompanied by progressive neuronal loss and cognitive impairment. Changes are caused by modifications in amyloid precursor protein (APP) breakdown, synthesis of the APP fragment amyloid β‐protein (Aβ), and hyperphosphorylated tau protein aggregation, all of which contribute to decreased synaptic strength and synapse loss.^4^ In the brain, synapses create crucial connections between nerve cells, allowing for the signal transduction required for cognitive functions. A range of signaling and chemical pathways play a role in the confluence of axonal and dendritic filopodia to create intercellular connections during synaptogenesis. A healthy brain requires synaptic development and plasticity, which, when dysregulated, can result in neurodegenerative disorders.

Synapses are asymmetric intercellular connections, mediated by cell adhesion molecules (CAMs), that allow neurons to communicate quickly and connect neurons to circuits.^5^ The primary function of CAMs is cross‐cellular signaling. Several synaptic CAMs that initiate synapse formation and maintain presynaptic and postsynaptic connections have been identified. For example, the human L1‐CAM has been found to be involved in disrupting synapse formation, resulting in neurological disorders.^6^ The N‐terminal domain of the protein tyrosine phosphatase receptor type O (PTPRO) has been demonstrated to be a synaptic CAM, which serves as an initiator for synapse formation.^7^ Synapse formation connects the presynaptic and postsynaptic sides. The synapse's presynaptic and postsynaptic components coordinate the precise arrangement of their localization and activation, allowing for both short‐ and long‐term synaptic transmission.^5^ Memory loss, difficulty in learning, difficulty in expressing emotions, and other typical symptoms of AD are caused by synaptic development problems. Therefore, a further in‐depth understanding of the molecular mechanisms that promote and sustain these synapses is required.

Wnt signaling is one of the most critical signaling systems that comprises evolutionarily conserved and entangled pathways. Cell adhesion and cell fate determination have been demonstrated to be critically and irreplaceably related to Wnt signaling.^8^, ^9^, ^10^ Wnt signaling is related to many severe diseases, including AD. Studies have revealed that loss of Wnt signaling or dysregulation of Wnt signaling triggers the pathogenesis of AD.^11^, ^12^, ^13^ This review summarizes the relationship between Wnt signaling and AD. Considering the regulation of Wnt signaling in synaptogenesis, this could lead to a better understanding of Wnt‐involved synaptogenesis, AD pathology, and novel research avenues.

## 2 WNT SIGNALING PATHWAY

**Figure 1:**
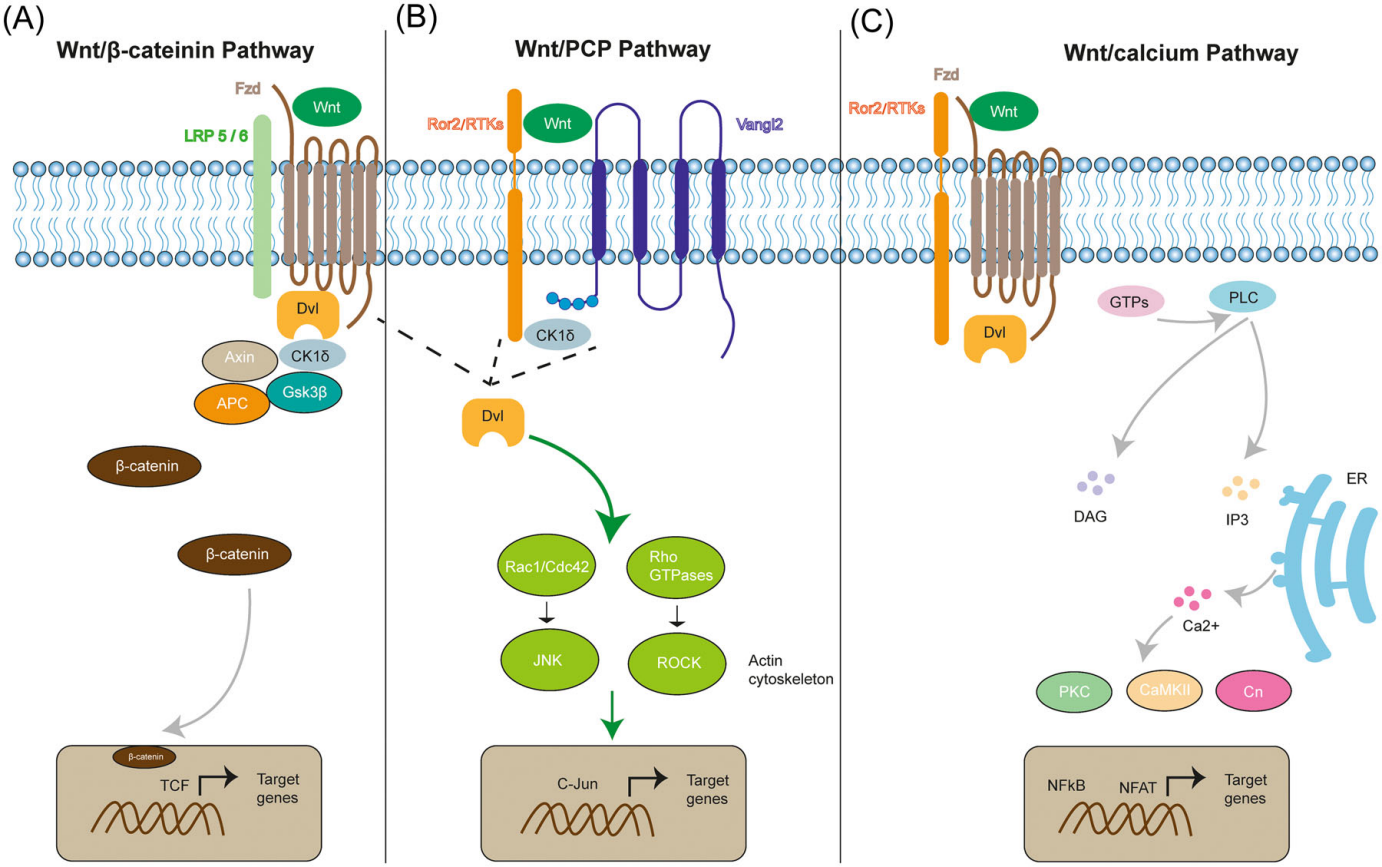
Wnt signaling pathway. (A) Wnt canonical Wnt/β‐catenin signaling pathway. Wnts bind with its receptor Fzds and coreceptor Lrp5/6, inactivating the destruction complex and accumulating β‐catenin in the cytosol. β‐catenin then translocates into the nucleus and activates downstream signaling. (B) Wnt noncanonical Wnt/PCP signaling pathway. Wnts bind with its receptor Ror2 in a Vangl2‐dependent fashion and then activate the target genes. C. Wnt noncanonical Wnt/Ca^2+^ signaling pathway. Wnts bind with its receptor Fzds in a Ror2/RTKs‐dependent manner, recruiting the Dvl and promoting the release of Ca^2+^. Active kinase proteins, then associate with NFkB and NFAT factors and activate the target genes. [Color figure can be viewed at wileyonlinelibrary.com]

Wnt represents a combination and is an abbreviation of the words *Wingless* and *Int‐1*.^14^ Wnt encodes lipid‐modified signaling glycoproteins with 350–500 amino acids that can be palmitoylated.^15^, ^16^ Due to this modification, Wnt has the properties of insolubility and hydrophobicity, making transportation of the Wnt morphogens more difficult. Wnt has three branches of pathways (shown in Figure 1): the canonical Wnt/β‐catenin, the noncanonical Wnt/planar cell polarity (PCP) pathway, and the Wnt/calcium pathway. For the Wnt/β‐catenin signaling pathway (Figure 1A), when the Wnt is absent, β‐catenin will form a destruction complex in the cytosol together with glycogen synthase kinase three beta (GSK3β), adenomatous polyposis coli (APC), and casein kinase‐1 (CK1). In turn, β‐catenin will then be phosphorylated by CK1 and GSK3β and degraded via a proteasome.^17^ When Wnt is present, the Wnt ligand will bind to its corresponding receptors (e.g., seven‐pass transmembrane receptor, Frizzled receptors, and coreceptor low‐density lipoprotein receptors). Then, the scaffolding protein Dishevelled will recruit Axin, CK1, APC, and GSK3β, promoting the accumulation of β‐catenin in the cytosol and allowing its translocation into the nucleus to activate the downstream genes through the T‐cell factor/lymphocyte enhancer factor (TCF/LEF) transcription factor.^17^, ^18^

One branch of the Wnt noncanonical pathway is the Wnt/PCP pathway (Figure 1B). It is essential for the regulation of cell proliferation and cytoskeletal rearrangements during development. The Wnt ligands (e.g., Wnt5 and Wnt11) bind to their receptors Frizzled or tyrosine kinase‐like orphan receptor 2 (Ror2), recruiting casein kinase I (CK1δ), and then phosphorylates the protein Van‐Gogh‐like 2 (Vangl2) in response to Wnt activation, resulting in the activation of Dishevelled (Dvl). Upon activation, the small GTPase Rho, Rac1, and Rho‐subfamily (such as Cdc42) work together to activate downstream JNK and ROCK signaling, facilitating polarized cell migration, proliferation, and actin cytoskeleton modification.^19^ Another branch is the Wnt/calcium pathway (Wnt/Ca^2+^) (Figure 1C). Wnt ligands bind to their Frizzled receptors and coreceptor Ror/Ryk, consequently recruiting and activating Dvl. The small GTPase then activates the plasma membrane phospholipase C (PLC), producing and increasing the concentrations of inositol 1,4,5‐triphosphate (IP3) and diacylglycerol (DAG).^20^ IP3 promotes intracellular Ca^2+^ release by interacting with the endoplasmic reticulum (ER).^21^ The intracellular signaling cascades are then activated, resulting in an increase in the concentration of calcium ions in the cytoplasm. Calcium‐dependent kinases such as protein kinase C (PKC), calcium calmodulin‐dependent protein kinase II (CaMKII), calpain 1, and calcineurin (Cn) are activated by increased Ca^2+^ and DAG. Active kinase proteins are typically related to the activation of downstream transcription factors such as nuclear factor associated with T cells (NFAT) and nuclear factor‐kappa B (NFkB).^20^, ^21^, ^22^ Wnt/Ca^2+^ signaling has been linked to several biological processes, such as control of gene expression, cell motility, differentiation, and proliferation.^23^

## 3 THE WNT SIGNALING IN AD

Wnt signaling is a highly conserved pathway that regulates several biological activities, including cell proliferation, differentiation, and cell fate determination. It has been linked to the onset of several diseases, including cancer and neurological disorders like AD.^24^ AD is characterized by the formation and accumulation of Aβ plaques and neurofibrillary tangles (NFTs) in the brain, which are made up of hyperphosphorylated tau protein, and triggers a cascade of pathogenic processes including neuroinflammation and synaptic dysfunction, which ultimately lead to neuronal death and cognitive loss. The specific mechanisms that cause AD are unknown; however, multiple studies have suggested that the Wnt signaling pathway plays a vital role in this pathogenesis.

Wnt signaling is important in controlling synaptic plasticity and neurogenesis in the brain, and changes in this system may contribute to the development of AD. Wnt signaling pathway activation has been found to have neuroprotective benefits by boosting neuron survival and differentiation and decreasing Aβ build‐up and tau hyperphosphorylation.^24^ On the other hand, Wnt signaling suppression has been linked to increased Aβ production and tau hyperphosphorylation, as well as synaptic dysfunction and neuronal death.^11^ Here, to be more specific, Wnt signaling is Wnt/β‐catenin signaling. For instance, inhibition of GSK3β, one of the most important regulators of β‐catenin, was reported to boost the accumulation of β‐catenin, allowing for the reduction of AD pathogenesis.^25^, ^26^ Aβ can mediate GSK3β activity and can induce cognitive impairment, resulting in fewer synapses and postsynaptic density (PSD) thickness loss.^27^ In terms of these defects, Wnt noncanonical pathways, which include Wnt/PCP and Wnt/Ca^2+^ pathways, have also been implicated. Wnt/PCP and Wnt β‐catenin are mutually repressive.^28^ AD‐related downregulation of Wnt β‐catenin upregulates Wnt/PCP signaling to some extent, which is related to synaptic plasticity and neuroinflammation. Wnt5a has been shown to modulate the presynaptic and postsynaptic structure and influence synaptic transmission, indicating that the Wnt/JNK signaling pathway regulates the formation and function of presynaptic and postsynaptic synapses.^29^, ^30^ Wnt5a can also activate the Wnt/Ca^2+^; signaling pathway by regulating CaMKII, thus affecting neurite growth.^31^

## 4 WNT SIGNALING IN SYNAPSES

The regulation of synaptogenesis involves critical signaling systems, notably the Wnt pathways (see the review in Dickins and Salinas^32^). Wnt ligands engage diverse receptors and associated molecules to mediate inter‐ and intracellular signal transmission. Wnt proteins are a class of secreted signaling molecules that influence synapse formation by governing the assembly of presynaptic and postsynaptic elements in central and peripheral synapses. The signaling pathways initiated by Wnts interact with distinct receptors such as Frizzleds (Fzds), the LRP5/6 coreceptors, Ror and Ryk (see the review in Sahores and Salinas^33^). Within the context of synapse formation, various Wnt proteins and pathways orchestrate crucial developmental mechanisms, including axon and dendrite guidance, target selection, maturation, and plasticity.

### 4.1 Axon and dendrite guidance

**Figure 2:**
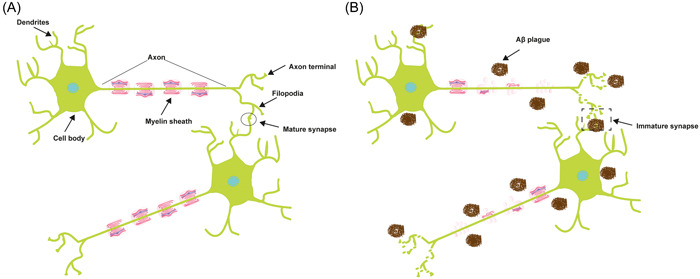
Anatomy of a neuron and neuron connection. (A) The cellular structure of a neuron includes a soma, which acts as the nucleus' core and gives rise to dendrites, which are responsible for receiving signals and are distinguished by their condensed and branching shape. In contrast, an axon, which is a long, thin projection covered in a myelin sheath, emerges from the opposing facet. The axon terminals, the furthest points of neuronal extension, are produced and transmit signals. (B) The structure of a neuron and neuron connection are disrupted by amyloid β‐protein (Aβ) plague. [Color figure can be viewed at wileyonlinelibrary.com]

Neurons are characterized as highly polarized cells with two distinct domains, arising from the cell body (Figure 2A). One of these domains comprises a solitary, elongated axon responsible for transmitting signals, while the other consists of numerous shorter dendrites that are specialized to receive signals.^34^ The myelin sheath protects the outside of an axon (Figure 2A). The myelin sheath comprises a protective, lipid‐rich encasement encompassing nerve fibers, akin to the insulating sheath around electrical wires. This enveloping structure facilitates the swift transmission of electrical impulses between nerve cells bidirectionally. In the event of myelin damage, the continuity of these electrical signals becomes disrupted, potentially leading to their complete cessation. In Figure 2, the relationship between a dendrite and an axon can be linked to that between receiving and sending. Dendrites have a complex branching morphology, which facilitates receipt of neuronal impulses from neighboring cells, and engage in an interpretation process to determine whether to pass the signals. Once determined, dendrites will generate an electrical impulse transmitted through the myelin sheath,^35^ while axons, characterized by their elongated and unbranched structure, transmit impulses away from the cell body (reviewed in Waxman^36^). With the progressive aggregation of Aβ plaques, the integrity and functionality of the myelin sheath become compromised, leading to impaired transmission of neural impulses. Consequently, there is disruption and disorganization in the structure of the axon terminal, leading to the formation of immature synapses (Figure 2B).

### 4.2 Structure and formation of dendrites

**Figure 3:**
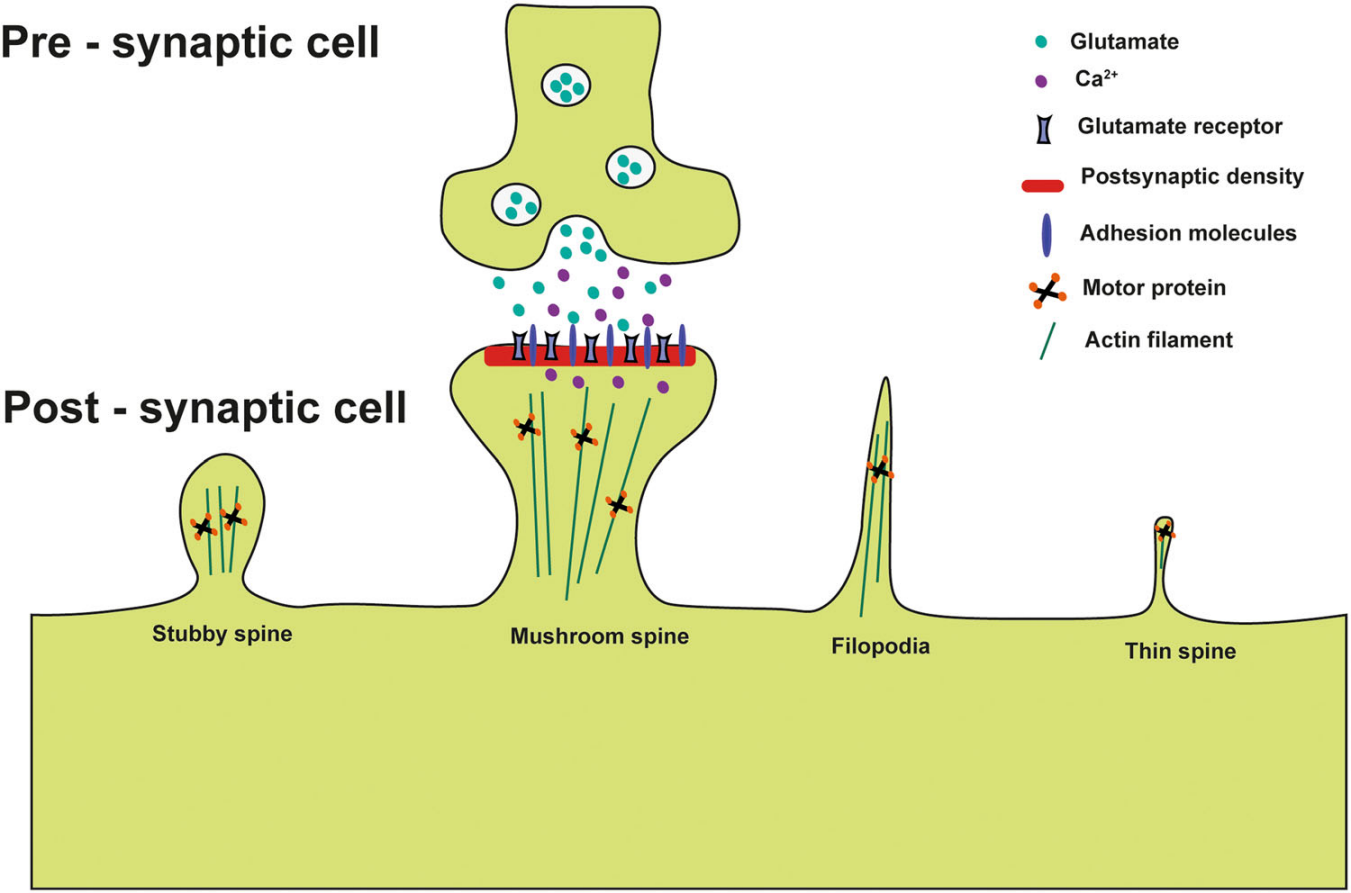
Schematic drawing of a synapse between presynaptic cells and various types of postsynaptic dendritic spines. [Color figure can be viewed at wileyonlinelibrary.com]

In neurons, cells have diverse intercellular structures, such as gap junctions and synapses, that facilitate cell–cell communication. Among these structures, cytonemes or filopodial bridges play a crucial role in connecting adjacent cells through adhesion mechanisms, allowing for the transfer of surface‐associated cargoes from one cell to another via ligand–receptor‐mediated interactions (Figure 3).^37^ In cytoneme‐mediated signaling and neuronal synaptogenesis, the process of the formation of membranous protrusions known as filopodia show significant similarities in their initial structural regulation and dynamics. These parallels include the involvement of actin‐based bundled and branched cytoskeletal elements.

While the identification of protrusions may involve some ambiguity, a distinctive characteristic of filopodia is their reliance on RhoGTPase Cdc42, which imparts a noteworthy unifying trait to both synaptic filopodia and cytonemes. The recruitment of Cdc42 to the plasma membrane and its interaction with N‐Wasp are initiating factors for filopodial formation through remodeling of the actin cytoskeleton.^38^ This process is crucial to the development of cytonemes and dendritic filopodia, which play a significant role in synapse signaling and maturation. However, in AD neurons, the dendrites are interrupted and do not function. Although the precise inhibition mechanisms in these dendrites remain unknown, the available evidence indicates that the inhibition or knockout of Cdc42 leads to dysregulated actin remodeling and reduced filopodial abundance, implying that comparable outcomes may be observed in filopodial synaptic precursors. This observation is particularly noteworthy considering the established importance of this RhoGTPase in synapse formation and plasticity.^39^

Cdc42 and RhoGTPase are important downstream factors in Wnt/PCP signaling. Research has shown that Wnt7a and Wnt7b, interacting with frizzled‐5 and frizzled‐7 receptors, play a crucial role in the regulation of dendritic spine growth and synaptic strength by locally activating CaMKII and JNK at dendritic spines. Consequently, any irregularities in Wnt7a/b signaling may contribute to the pathogenesis of neurological disorders characterized by disrupted excitatory signaling.^40^, ^41^, ^42^ Wnt β‐catenin signaling is also involved in regulating synaptic filopodia and cytonemes. Research has shown that Wnt8a, together with Ror2, are essential for filopodial growth. Upon activation, Ror2 initiates intracellular signaling cascades that initiate the assembly of cytonemes. The degree of Ror2 activation correlates positively with the number of cytonemes formed by the cell.^28^ β‐catenin augmentation induces the expansion of dendritic structures and their branching patterns by facilitating or stabilizing short dendritic branches.^43^

### 4.3 The morphology of dendrites

The advent of novel imaging methodologies has enabled the examination of the intricate associations between the structural characteristics of dendrites and their functional attributes. The dendritic spine constitutes a bulbous projection stemming from the dendritic shaft, frequently linked by a slender neck that gives rise to a distinct spine head (as shown in Figure 3). Situated at the extremity of the spine head in proximity to the presynaptic terminal, the PSD is notably abundant in receptors, proteins, and signaling molecules^44^ (as shown in Figure 3). As shown in Figure 3, the presynaptic terminal facilitates the exocytosis of glutamate neurotransmitters and associated signaling molecules. Subsequently, these bioactive compounds engage in receptor‐mediated interactions within the adjacent postsynaptic cells, thereby initiating cascades of downstream intracellular signaling events.

Dendritic spines have a range of morphological variations. Consequently, initial investigations categorized these structures into three distinct types: (1) spines characterized by elongated, slender necks and diminutive heads, termed “thin spines,” (2) spines possessing wider necks and prominent heads, referred to as “mushroom spines,” and (3) abbreviated spines lacking delineated necks, denoted as “stubby spines.”^45^ The dendritic spine formation takes precedence over synaptogenesis within the adult brain, constituting a fundamental stage in neuronal maturation and thereby exerting a pivotal influence on processes such as learning and memory. Notably, the configurations characterized by mushroom and stubby morphologies show heightened stability and prevalence within mature synapses, facilitating the establishment of enduring connections that are integral to memory retention.^46^ Conversely, the elongated and slender protrusions manifest heightened dynamic instability, indicating their potential involvement in more transient synaptic dynamics.

Nonetheless, the prevailing perspective posits that a filopodia‐like extension originating from the dendritic shaft may establish a connection with the axon, initiating the synaptogenesis process. This nascent connection is believed to develop into a slender spine, which undergoes maturation to transition into the mature morphology characteristic of a mushroom spine (reviewed in Bonilla‐Quintana and Rangamani^44^). Evidence has indicated that the prevalence of filopodia‐like extensions originates from dendritic structures. These extensions show a dynamic behavior, characterized by elongation and retraction over hours. Such structural features are particularly abundant during the early stages of animal development and subsequently evolve to attain heightened stability within the mature phase. This phenomenon offers a plausible anatomical foundation that underlies the potential for sustained storage of informational content over extended periods.^47^

## 5 SYNAPSE IN AD

Synapses represent the connections between the axons and dendrites of neuronal cells within the brain and the central nervous system.^48^ Their function involves the controlled and directional transmission of chemical^49^ and electrical signals,^50^ eliciting various responses. This rapid signal transduction between cells is crucial for immediate cognitive functions like decision‐making. Synaptogenesis encompasses various stages, including growth, stabilization, maturation, maintenance, and controlled degradation of the presynaptic and postsynaptic components of neuronal cells. This intricate process relies on numerous genes, proteins, and receptor–ligand interactions.^51^ Synaptic plasticity, which involves physiological changes and strengthening of synaptic components due to prolonged excitation, has been associated with memory and learning.^52^ On the other hand, the loss of synapses has been linked to neuropathological conditions like AD.

AD is marked by several prevalent attributes, including synaptic abnormalities and dysfunctions, such as synaptic damage, synaptic loss, and structural modifications in the synapse (reviewed in Peng et al.^53^). Dendritic spines, minute protrusions distributed along dendrites, serve as prominent postsynaptic loci facilitating excitatory synaptic transmission. These spines have the remarkable capability of plasticity and can be modified, even in the mature nervous system. Spine remodeling and synapse formation are reliant on activity, constituting a fundamental foundation for memory establishment. Perturbation or modification of these structures has been reported in individuals afflicted with neurodegenerative conditions like AD (reviewed in Knobloch and Mansuy^54^). These irregularities manifest during the initial phases of the disease, preceding the onset of behavioral symptoms. Evidence has shown that Aβ oligomers possess the capacity to interfere with or perturb synaptic efficacy and plasticity.^55^, ^56^ Soluble Aβ oligomers, existing at low concentrations within the context of AD brain, evoke heightened neuronal excitability through their disruptive impact on the delicate equilibrium between glutamatergic and GABAergic signaling pathways, consequently leading to impaired synaptic plasticity.^57^ Research involving soluble Aβ oligomers derived from the brains of individuals with AD indicates that specific bioactive configurations, particularly small and diffusible oligomers, possess the capacity to impede synaptic plasticity.^58^ This disruptive effect is achieved through diverse mechanisms, such as engaging plasma membranes and altering the balance of excitatory and inhibitory neurotransmission, perturbing metabotropic glutamate receptors (mGluR), prion protein (PrP), and other neuronal surface proteins, reducing the expression of glutamate transporters, inducing glutamate spillover, and activating extrasynaptic N‐methyl‐d‐aspartate (NMDA) receptors containing GluN2B subunits.^58^ Consequently, understanding the underlying mechanisms responsible for synaptic dysfunction in AD and focusing on synaptic interventions, particularly during early treatment stages, has the potential to unveil innovative and enhanced therapeutic approaches capable of ameliorating the quality of life of individuals afflicted with AD.

## 6 WNT‐TARGETING PREVENTION AND THERAPIES FOR AD

A prominent cause of morbidity among the elderly, age‐related diseases, notably AD, has been the focus of extensive research efforts dedicated to investigating potential therapeutic interventions and preventive strategies. However, finding a permanent cure requires a thorough understanding of the underlying molecular mechanisms governing the disease pathology. This knowledge can serve as the foundation for the development of targeted interventions to mitigate the symptomatic manifestations associated with the disorder. At present, the prevailing pharmacological approaches predominantly involve the administration of cholinesterase inhibitors and a NMDAR (N‐methyl‐d‐aspartate receptor) antagonist (reviewed in Farlow^59^). Despite their utilization, the efficacy of these interventions is constrained, and at the same time, the appearance of side effects reduces the scope of their use in the patient population. Therefore, a greater understanding of the complex molecular foundations related to the condition can considerably enhance the landscape of AD research.

The activation of the Wnt pathway, by repression of factors implicated in Toll pathway activation, has demonstrated the capacity to ameliorate the deleterious outcomes associated with amyloidogenesis and oligomerization, concurrently exerting inhibitory effects on inflammation within a designed optogenetic paradigm utilizing *Drosophila* gut stem cells.^60^ These findings imply the prospect of using pharmaceutical agents that activate the Wnt pathway for potential therapeutic interventions in AD. DKK1, as the Wnt inhibitor, is induced by the presence of Aβ, which perturbs the canonical Wnt/β‐catenin signaling pathway and enhances the noncanonical Wnt signaling pathways. The Wnt/β‐catenin signaling pathway plays a significant role in maintaining synaptic stability, whereas the noncanonical Wnt signaling pathway promotes the retraction of synapses. In this manner, the loss of synapses induced by Aβ can be modulated through the involvement of DKK1. Elliott et al. have reported that pharmacological intervention targeting the Aβ‐Dkk1‐Aβ positive feedback loop by administering the medication fasudil can potentially restore equilibrium within the Wnt signaling pathways. Using a mouse model characterized by progressive amyloid disease, this intervention is successful in preventing dendritic spine retraction in vitro and decreasing the accumulation in vivo.^61^ Marzo et al.^62^ investigated the effects of the Wnt antagonist Dkk1 on the adult rat hippocampus. Their research identified instances of synapse loss as well as changes in synaptic plasticity and impairments in long‐term memory. Furthermore, the authors revealed that stopping Dkk1 expression triggered synapse regeneration, which coincided with the restoration of long‐term memory function. This enhanced understanding has the potential to aid in the development of treatments aimed at reducing clinical symptoms while also improving our understanding of the fundamental mechanisms governing memory and learning processes.

## 7 CONCLUSION

According to research, synaptogenesis requires the activation of a wide array of signaling pathways to promote the proliferation, directional guidance, and stabilization of interneuronal connections required for the normal processes of cognition, memory, and learning. The Wnt family, which is delivered to specific locations via signaling filopodia, also called cytoneme, is notable among these signaling molecules. The structural and functional molecular mechanisms governing cytonemes and synaptogenic dendritic filopodia show substantial similarities, implying that these protrusions are part of a common system whose efficiency varies depending on the signaling milieu.

Changes in filopodia stability have a negative impact on synapse formation in neurodegenerative disorders such as AD, resulting in impaired cognitive capacities. Furthermore, the number and shape of dendritic filopodia increase in the context of Alzheimer's disease. Improving our understanding of the molecular mechanisms underlying or contributing to AD holds promise for improving treatment strategies and laying the groundwork for future research into novel pharmacological targets. However, the implementation of such approaches poses significant problems. This could include boosting Wnt factors that rely on β‐catenin or decreasing PCP‐dependent factors that maintain homeostasis. The precision of therapy application must be limited to neurons only, preventing misalignment of Wnt signaling in other somatic tissues, which could lead to the onset of carcinogenic processes. This is significant since abnormal levels of Wnt signaling, both increased and decreased, have been linked to the development of a variety of cancers. On the other hand, altering Wnt/β‐catenin signaling activity appears to be an attractive avenue for therapeutic intervention; the effort to develop pharmacological agents, either as activators or inhibitors, is hampered by difficulties in achieving optimal safety profiles and the desired selectivity.^63^

As a result, further research validating the relationship between Wnt and dendritic filopodia could lead to a new field of collaborative investigation and propel advancements in Alzheimer's research while broadening the scope of understanding neuronal regulation in general. Nonetheless, evidence of dissimilarity across these filopodia would highlight areas of divergence accessible to targeting cell‐specific processes, expanding the evolving wealth of knowledge about synaptogenesis and signal transit.

## AUTHOR CONTRIBUTIONS

Cheng‐Ting Zhang and Wen‐Yuan Wang conceptualized this review and wrote the manuscript. Joy Wang revised the manuscript.

## CONFLICT OF INTEREST STATEMENT

Cheng‐Ting Zhang is an editorial member of Ibrain and Wen‐Yuan Wang is the Associate editor of Ibrain; they are co‐authors of this article. They were excluded from editorial decision‐making related to the acceptance and publication of this article. The authors declare no conflicts of interest.

## ETHICS STATEMENT

Not applicable.

## Data Availability

The authors have nothing to report.
